# Editorial: Predictive biomarkers to immune checkpoint inhibitors in lung cancer

**DOI:** 10.3389/fimmu.2026.2011872

**Published:** 2026-10-20

**Authors:** Alessandro Russo, Christian Rolfo, Mariacarmela Santarpia

**Affiliations:** 1 Department of Biomedical Sciences, Humanitas University, Pieve Emanuele, Milan, Italy; 2 Medical Oncology Department, Humanitas Istituto Clinico Catanese, Misterbianco, Catania, Italy; 3 Department of Internal Medicine, Division of Medical Oncology, The Arthur G. James Comprehensive Cancer Center, Columbus, OH, United States; 4 Division of Medical Oncology, “Gaetano Martino” Hospital, Messina, Italy; 5 Department of Human Pathology “G. Barresi”, University of Messina, Messina, Italy

**Keywords:** immunotherapy, liquid biopsy, NSCLC, PD-L1, predictive biomarker

Immune checkpoint inhibitors (ICIs) have revolutionized the therapeutic landscape of lung cancer with unprecedented survival gains in the majority of clinical settings. After initial signs of effectiveness in pretreated non-small cell lung cancer (NSCLC) in the early 2010s (1, 2), the use of ICIs has progressively changed the standard of care in first-line metastatic settings, locally advanced disease, and, more recently, early-stage NSCLC (3, 4). However, except for programmed death ligand 1 (PD-L1) tumor expression (tumor proportion score, TPS), due to the inherent biological limitations of this biomarker, no other predictive factor has been approved to guide patient selection for these agents (5), posing several challenges in clinical practice. Furthermore, the use of ICIs in small cell lung cancer (SCLC), whether in the extensive stage or limited stage of the disease, is independent of PD-L1 status or any other predictive biomarker (6). The identification of predictive biomarkers is essential to reducing the cost of these treatments and avoiding unnecessary toxicities in patients who cannot benefit from these drugs. In this rapidly evolving therapeutic scenario, it is crucial to understand the basis of tumor response and resistance to drive a more rational development of novel immunotherapy-based strategies.

The Research Topic *“Predictive Biomarkers to Immune Checkpoint Inhibitors in Lung Cancer”* aimed to identify novel, reliable predictive biomarkers for ICIs that could drive therapeutic decision-making in lung cancer more effectively. The clinical and scientific relevance of the topic is further highlighted by the success of this collection, with 19 published articles, including 9 original research studies, 5 systematic reviews, 3 literature reviews, and 2 case reports. More than 130 authors from different countries contributed to this special issue.

Circulating biomarkers are particularly appealing in this context, due to their minimally invasive nature and, in some cases, low evaluation cost. Examples include the derived neutrophil-to-lymphocyte ratio (dNLR), lactate dehydrogenase (LDH), and the Lung Immune Prognostic Index (LIPI) score. Their prognostic value is well known (7) and was further explored in some of the research studies presented here, including in largely underexplored settings, such as SCLC. In addition to the prognostic value of these circulating biomarkers, others have explored their potential use in serial assessments of laboratory results, such as the absolute lymphocyte count, or their correlation with immunosuppressive circulating cytokine levels.

Growing evidence suggests a pivotal role of the microbiome in ICI response within solid tumors, including lung cancer. The microbiome may be influenced by concomitant therapies, such as antibiotics or proton pump inhibitors (PPIs) (8). The microbiome represents a potential therapeutic target in patients treated with ICIs, and fecal transplantation has been reported to potentially impact their therapeutic efficacy (9).

Overall, this Special Issue contributes to the significant efforts made in the last few years to understand ICI response in lung cancer, adding another piece to the complex puzzle of immunotherapy effects in solid tumors. The articles included in this Research Topic reflect the field’s rapid progress, moving beyond the conventional PD-L1-centric model toward a more comprehensive view of the complex interactions between the immune system and lung cancer. Future challenges will be to combine and integrate several layers of information, from tumor genomics and transcriptomics to liquid biopsies, the tumor microenvironment, and the microbiome (**Figure 1**), and validate them in prospective studies.

**Figure 1:**
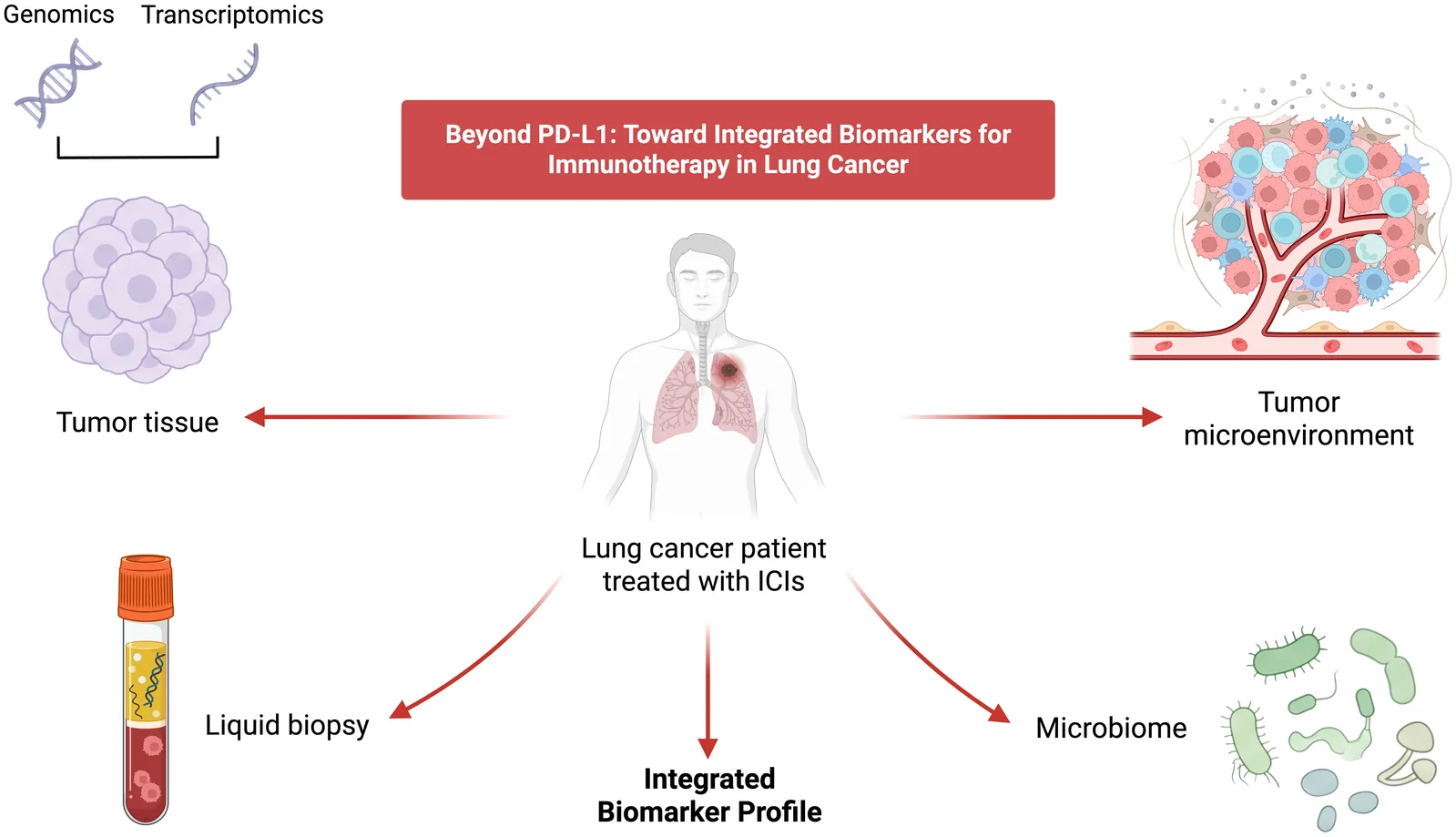
Beyond PD-L1: toward integrated biomarkers for immunotherapy in lung cancer. Created with BioRender.com.
